# Ischemic pancreatitis following uterine inversion and severe postpartum hemorrhage: A case report and review of the literature

**DOI:** 10.1016/j.crwh.2025.e00741

**Published:** 2025-08-07

**Authors:** Arisa Kodama, Yoshitsugu Chigusa, Yukio Yamanishi, Akira Korenaga, Yusuke Sakura, Haruta Mogami, Masaki Mandai, Takaaki Yoshida

**Affiliations:** a Department of Obstetrics and Gynecology, Japanese Red Cross Society Wakayama Medical Center, 4-20 Komatsubara-dori, Wakayama 640-8558, Japan; b Department of Gynecology and Obstetrics, Graduate School of Medicine, Kyoto University, 54 Shogoin Kawahara-cho, Sakyo-ku, Kyoto 606-8507, Japan; c Department of Emergency and Intensive Care Medicine, Japanese Red Cross Society Wakayama Medical Center, 4-20 Komatsubara-dori, Wakayama 640-8558, Japan; d Department of Gastroenterological Surgery, Japanese Red Cross Society Wakayama Medical Center, 4-20 Komatsubara-dori, Wakayama 640-8558, Japan

**Keywords:** Uterine inversion, Postpartum hemorrhage, Ischemic pancreatitis, Cardiac arrest, Multidisciplinary management

## Abstract

Ischemic pancreatitis is a rare but potentially life-threatening condition typically associated with cardiovascular events, such as aortic dissection and cardiogenic shock. This report presents a unique case of ischemic pancreatitis following severe postpartum hemorrhage (PPH) and cardiac arrest caused by uterine inversion. A 34-year-old woman developed uterine inversion immediately after delivery, which resulted in massive hemorrhage and cardiac arrest. The patient was stabilized in the intensive care unit after successful resuscitation and uterine repositioning. On postpartum day 5, the patient developed fever and hypotension. On postpartum day 9, contrast-enhanced computed tomography (CT) revealed pancreatic enlargement and peripancreatic fluid collection consistent with acute pancreatitis. The absence of common etiologies, such as alcohol use, gallstones, or hypertriglyceridemia, along with a clear temporal relationship between hemorrhagic shock and cardiac arrest, strongly supported a diagnosis of ischemic pancreatitis. Despite initial conservative treatment and endoscopic drainage, the persistent fever necessitated open surgical drainage. The patient recovered completely and was discharged on postpartum day 89. This appears to be the first case report to provide a detailed description of the clinical course and a therapeutic strategy for ischemic pancreatitis following PPH. Clinicians should recognize that ischemic pancreatitis may develop as a secondary complication in patients with PPH complicated by cardiac arrest. If pancreatitis is suspected, prompt contrast-enhanced CT and timely multidisciplinary management are essential to achieve an accurate diagnosis and initiate effective treatment.

## Highlights

• This appears to be the first detailed report of ischemic pancreatitis secondary to postpartum hemorrhage.
• Cardiac arrest with postpartum hemorrhage may trigger ischemic pancreatitis.
• Computed tomography for diagnosis and multidisciplinary treatment are crucial for clinical management.

## 1 Introduction

Postpartum hemorrhage (PPH) remains a leading cause of maternal mortality, accounting for approximately 27 % of maternal deaths worldwide [1,2]. The etiologies of PPH include uterine atony, genital tract trauma, retained placenta, and abnormal placental implantation [2,3]. Although rare, acute puerperal uterine inversion is particularly concerning because of its potential to cause rapid massive hemorrhage and hypovolemic shock [4]. Without prompt and appropriate intervention, maternal death can occur, rendering it an obstetric emergency.

Massive hemorrhage and ensuing hypovolemic shock can result in systemic hypoperfusion and ischemic injury to multiple organs. In obstetrics, Sheehan's syndrome (postpartum pituitary necrosis) is a well-recognized example of such an injury [5]. Additionally, case reports have described PPH-induced ischemic complications, including renal cortical necrosis [6] and ischemic hepatitis [7,8], further underscoring the potential for widespread organ dysfunction in this setting.

The pancreas is also known to be highly sensitive to ischemia, and ischemic pancreatitis has been reported in non-obstetric scenarios, particularly in association with massive hemorrhage secondary to aortic dissection, trauma, and other causes [9]. In a published case series of ischemic pancreatitis, only two cases were noted to have occurred following PPH, but no clinical details were provided [9]. Here, we present an extremely rare and well-documented case of severe ischemic pancreatitis that developed immediately after delivery after massive postpartum hemorrhage and cardiac arrest due to uterine inversion, which ultimately required surgical intervention.

## 2 Case Presentation

The patient was a 34-year-old multiparous woman (gravida 3, para 2) who underwent laparoscopic left ovarian cystectomy at 24 years of age. She conceived through artificial insemination at another clinic and was referred to hospital at 27 weeks of gestation wishing to deliver at the same institution. The pregnancy progressed uneventfully. She was admitted to the hospital at 38 weeks and 0 days of gestation with spontaneous rupture of membranes and labor onset and delivered a healthy male infant vaginally on the same day. The Apgar scores were 9 and 10 at 1 and 5 min, respectively.

**Fig. 1:**
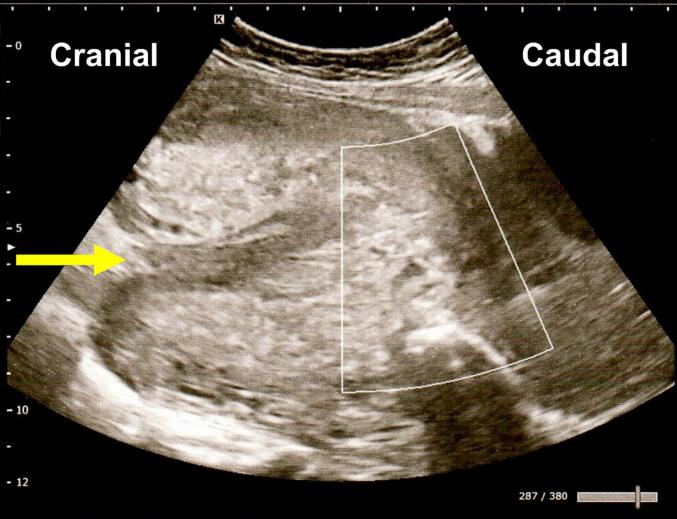
Sagittal transabdominal ultrasound image during uterine inversion. The fundal depression caused by the inversion (arrow).

The placenta did not separate immediately after delivery; however, spontaneous expulsion occurred after 1 h 22 min without manual or surgical intervention. Immediately after the placental expulsion, the patient complained of severe lower abdominal pain and presented with severe vaginal bleeding. Her blood pressure decreased to 74/43 mmHg, and her heart rate increased to 139 beats/min. A pelvic examination revealed an inverted uterine fundus within the vaginal canal. Transabdominal ultrasonography confirmed a diagnosis of uterine inversion (Fig. 1). Manual repositioning of the uterus was performed approximately 20 min after placental delivery under intravenous ritodrine infusion and intramuscular pentazocine for pain control. Although the uterus was initially successfully repositioned, the genital bleeding and abdominal pain worsened shortly thereafter, and uterine inversion reoccurred. At this point, the total estimated blood loss was 3000 mL. Blood transfusion was initiated and the decision was made to attempt repositioning of the uterus under general anesthesia.

However, the patient subsequently experienced cardiac arrest, and chest compressions were immediately initiated along with bag-valve-mask ventilation. Central venous access was established and rapid blood transfusion and epinephrine administration were initiated. After approximately 40 min of continuous cardiopulmonary resuscitation, the anesthesiologist successfully performed endotracheal intubation. The patient's pulse rate returned to approximately 50 beats/min, and chest compressions were discontinued. Under general anesthesia, uterine inversion was subsequently corrected. An intrauterine balloon was inserted and uterotonic agents were administered to prevent inversion recurrence. The total estimated blood loss was approximately 5300 mL. The patient received a transfusion of 14 units of red blood cells, 18 units of fresh frozen plasma, and 3 g of fibrinogen.

**Fig. 2:**
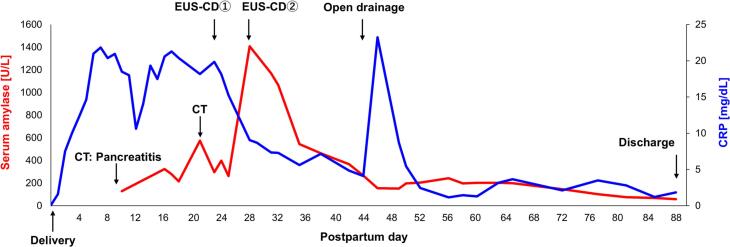
Clinical course of the present case. The serum amylase (red line) and C-reactive protein levels (blue line). CT, computed tomography, EUS-CD: endoscopic ultrasound-guided cyst drainage. (For interpretation of the references to colour in this figure legend, the reader is referred to the web version of this article.)

**Fig. 3:**
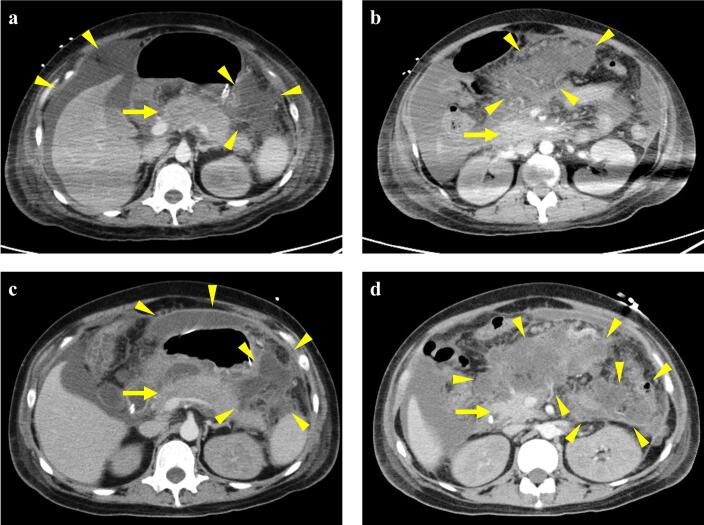
Contrast-enhanced computed tomography images. (a, b). On postpartum day 9, the pancreas appears enlarged (arrow), with surrounding fat stranding and peripancreatic and mesenteric fluid collection (arrowhead), which suggest acute pancreatitis. (c, d). On postpartum day 21, pancreatic enlargement (arrow) persisted without significant changes. Increased encapsulated peripancreatic fluid collections (arrowhead), with areas of internal heterogeneity suggestive of evolving necrosis, consistent with acute necrotic collections.

After uterine repositioning, the patient was closely monitored in the intensive care unit. The clinical course of the patient is shown in Fig. 2. On postpartum day 5, she developed a high-grade fever of 38.8 °C and hypotension. She was diagnosed with septic shock, and treatment was initiated with norepinephrine, vancomycin, and meropenem. All indwelling catheters were replaced; however, fever persisted despite these interventions. On postpartum day 9, contrast-enhanced computed tomography (CT) was performed to evaluate the source of the infection (Fig. 3a and b). Imaging revealed a large volume of ascitic fluid and diffuse pancreatic enlargement. Additionally, inflammatory stranding and fluid collection were observed around the pancreas and mesentery, suggesting acute pancreatitis. Clinically, the patient had marked tenderness in the upper abdomen and increased serum pancreatic amylase levels. Initial management included conservative therapy, including broad-spectrum antibiotics, therapeutic paracentesis, and enteral nutrition.

On postpartum day 23, follow-up contrast-enhanced CT revealed encapsulated necrotic material in the peripancreatic region (Fig. 3c and d). Endoscopic ultrasound-guided cyst drainage (EUS-CD) was performed twice. Although temporary defervescence was achieved, fever recurred and residual walled-off necrosis was noted on the anterior surface of the pancreas. Therefore, open surgical drainage was performed on postpartum day 43. The postoperative course was uneventful, and the patient was discharged on postpartum day 89 (postoperative day 46) without any significant long-term complications, including neurological or cardiac sequelae.

## 3 Discussion

The report describes a case of ischemic pancreatitis that developed after massive PPH and cardiac arrest caused by uterine inversion. The patient experienced a life-threatening hemorrhage immediately after delivery and subsequently developed severe pancreatitis requiring surgical intervention. Nevertheless, she recovered fully following a multidisciplinary treatment approach. Although there are a few reports of ischemic pancreatitis following PPH in the literature, this appears to be the first to provide a detailed clinical description and therapeutic course in such a case.

Acute puerperal uterine inversion, with an incidence of approximately 2.9 per 10,000 deliveries [4], is a rare but critically dangerous obstetric complication requiring urgent intervention. The known risk factors include placenta accreta, abnormal placental implantation, and prolonged labor [4,10]. However, as in the present case, uterine inversion can occur in patients without any identifiable predisposing conditions. Here, the uterine fundus prolapsed through the cervical os into the vaginal canal, corresponding to a second-degree inversion, which was promptly diagnosed through pelvic examination and transabdominal ultrasonography. Even in more severe cases, such as fourth-degree inversion, in which the uterus and vaginal walls are completely externalized, the diagnosis can be delayed if the condition misdiagnosed, particularly by less experienced obstetricians.

Uterine inversion is a significant risk factor for PPH, with an odds ratio of 16.89 (95 % confidence interval [CI]; 1.62–176.12) for causing hemorrhage >1500 mL [11]. Presently, despite early diagnosis, the total estimated blood loss reached 5300 mL. Prompt initiation of intensive multidisciplinary management, including close coordination with physicians responsible for systemic care, is essential upon diagnosis. In the present case, intravenous ritodrine was administered to facilitate uterine relaxation during manual repositioning, reflecting its continued widespread use for tocolysis in Japan. However, ritodrine is known to carry cardiovascular risks. Safer alternatives, such as nitroglycerin or attempting manual repositioning under general anesthesia from the outset, might have been considered in this situation. Furthermore, intrauterine balloon tamponade and adequate administration of uterotonic agents are recommended to prevent recurrence after successful repositioning. Retrospectively, the balloon tamponade should have been inserted immediately following the initial repositioning.

Although acute pancreatitis is relatively common, ischemic pancreatitis is rare. Here, the patient had no history of alcohol use, gallstone disease, or hypertriglyceridemia, which are common etiologies of pancreatitis. Considering the absence of these predisposing factors and clear temporal association between massive postpartum hemorrhage and cardiac arrest, an ischemic mechanism was considered the most plausible cause. The prolonged hospitalization of 89 days in this case was largely attributable to guideline-recommended, stepwise management of severe acute pancreatitis. Initial conservative therapy was followed by minimally invasive interventions, and open surgical drainage was performed only after these measures proved insufficient. Furthermore, open drainage procedures are typically delayed until at least four weeks after disease onset to allow necrotic collections to mature, and prolonged inpatient care was required postoperatively to carefully monitor drainage effectiveness and gradually remove the drains.

A PubMed search using the terms “ischemic pancreatitis” or “ischemic acute pancreatitis” yielded 49 publications, of which only 18 were English-language case reports or case series involving 33 patients (23 [70 %] men, median age; 57 [20–90] years) (Table 1) [9,[12], [13], [14], [15], [16], [17], [18], [19], [20], [21], [22], [23], [24], [25], [26], [27], [28]]. The most frequently reported underlying cause was aortic dissection (10 cases), followed by cardiogenic shock (four cases) and cardiopulmonary bypass (two cases). In the 17 cases in which the time to onset was described, the median interval between the precipitating ischemic event and diagnosis of pancreatitis was two days (range 1–21 days). Regarding treatment, 25 of the 33 patients (75.8 %) were managed conservatively, whereas seven patients (21.2 %) required surgical intervention. In the present case, conservative management was initially attempted in combination with two sessions of EUS-CD; however, owing to persistent fever, open surgical drainage was ultimately required. Although the patient achieved a favorable outcome, 13 of 33 cases (39.4 %) reported in the literature resulted in death. This high mortality rate likely reflects the severity of the underlying conditions, and publication bias favoring the reporting of more severe cases may be a contributing factor.

**Table 1:** Summary of reported cases of ischemic pancreatitis in the English literature.

Author	Year	Age	Sex	Triggering event	Time to pancreatitis (days)	Treatment	Outcome
Abramowitz BR [12]	2025	60	M	Cardiogenic shock	Unknown	Conservative	Death
Fujieda M [13]	2025	52	M	Aortic dissection	5	Conservative	Alive
Huanggu H [14]	2023	37	M	Aortic dissection	1	Conservative	Alive
Einsfeld L [15]	2022	38	M	Heart transplantation	5	Conservative	Death
Tsomidis I [16]	2021	80	M	Aortic thrombus	Unknown	Conservative	Alive
Masuda S [17]	2020	72	M	Cardiopulmonary bypass	2	Endoscopic drainage	Alive
Nemoto M [18]	2019	71	M	Aortic dissection	15	Conservative	Alive
Wang R [19]	2018	63	F	Aortic dissection	2	Conservative	Alive
		48	M	Aortic dissection	Unknown	Conservative	Alive
		48	F	Aortic dissection	Unknown	Conservative	Alive
		46	F	Aortic dissection	13	Conservative	Alive
		68	F	Aortic dissection	2	Conservative	Alive
		54	M	Aortic dissection	4	Conservative	Death
Chua WM [20]	2017	90	F	Arterial embolization	1	Conservative	Death
Cocota I [21]	2015	68	M	Partially thrombosed aortic aneurysm	1	Conservative	Alive
Hamamoto M [22]	2012	47	M	Aortic dissection	1	Conservative	Alive
Piton G [23]	2010	58	M	Cardiac arrest	1	Conservative	Death
Vyas A [24]	2009	20	F	Thrombosis	Unknown	Conservative	Alive
Mast JJ [25]	2009	57	M	Dehydration (marathon)	2	Conservative	Alive
Hackert T [9]	2009	36	M	Cardiopulmonary bypass	Unknown	Surgery	Death
		65	M	Heart transplantation	Unknown	Surgery	Alive
		69	M	Ruptured aortic aneurysm	Unknown	Surgery	Death
		49	M	Septic shock	Unknown	Surgery	Death
		78	M	Cardiogenic shock	Unknown	Surgery	Death
		76	F	Vessel occlusion	Unknown	Conservative	Death
		65	M	Hypovolemic shock	Unknown	Conservative	Alive
		69	M	Vessel occlusion	Unknown	Surgery	Death
		43	M	Vessel occlusion	Unknown	Conservative	Alive
		30	F	Hypovolemic shock (PPH)	Unknown	Conservative	Alive
		25	F	Hypovolemic shock (PPH)	Unknown	Surgery	Death
Addario L [26]	2008	62	M	Arterial embolization	21	Conservative	Death
Bazuro GE [27]	2004	53	F	Cardiogenic shock	1	Conservative	Alive
Deviere J [28]	1987	29	M	Ergotamine poisoning (suicide attempt)	2	Conservative	Alive

M: male, F: female, PPH: postpartum hemorrhage.

A notable feature of the present case was that the patient experienced cardiac arrest secondary to PPH. PPH occurs in approximately 4.3 % of all deliveries and is therefore not uncommon in obstetric practice [29]. According to data from high-volume centers in Japan, the median blood loss in such cases is approximately 2800 mL [3], and hypovolemic shock is frequently observed. Nevertheless, reports on pancreatitis developing as a complication of PPH are extremely limited. The literature review has shown that most reported cases of ischemic pancreatitis follow cardiovascular events, such as aortic dissection or cardiogenic shock. Hence, it is reasonable to consider that, in the present case, the development of ischemic pancreatitis was triggered not solely by PPH but also by the profound circulatory collapse associated with cardiac arrest and subsequent resuscitation.

## 4 Conclusion

In cases of severe PPH resulting in cardiac arrest, clinicians should be aware of the potential for secondary complications, such as ischemic pancreatitis. Even after successful resuscitation, careful monitoring should be continued, including evaluation of upper abdominal pain and serum amylase levels. If pancreatitis is suspected, contrast-enhanced CT must be performed immediately for diagnosis and to initiate appropriate multidisciplinary treatment.

## Contributors

Arisa Kodama contributed to the patient care, the conception of the case report, data acquisition, manuscript drafting, and critical revision of the manuscript for important intellectual content.

Yoshitsugu Chigusa contributed to the conception of the case report, drafting the manuscript, and critical revision of the article for important intellectual content.

Yukio Yamanishi contributed to the patient care, the conception of the case report, data acquisition, manuscript drafting, and critical revision of the manuscript for important intellectual content.

Akira Korenaga contributed to the patient care, data acquisition, manuscript drafting, and critical revision of the article for important intellectual content.

Yusuke Sakura contributed to the patient care, data acquisition, manuscript drafting, and critical revision of the article for important intellectual content.

Haruta Mogami contributed critically to the revision of the article for important intellectual content.

Masaki Mandai contributed critically to the revision of the article for important intellectual content.

Takaaki Yoshida contributed to the patient care, data acquisition, manuscript drafting, and critical revision of the article for important intellectual content.

All authors approved the final submitted manuscript.

## Patient consent

Written informed consent was obtained from the patient for the publication of this case report and accompanying images.

## Provenance and peer review

This article was not commissioned and was peer reviewed.

## Funding

This study was supported by a grant from the 10.13039/501100004330Smoking Research Foundation in Japan (2023G021).

## Declaration of competing interest

The authors declare that they have no competing interest regarding the publication of this case report.
